# Dietary Chromium Restriction of Pregnant Mice Changes the Methylation Status of Hepatic Genes Involved with Insulin Signaling in Adult Male Offspring

**DOI:** 10.1371/journal.pone.0169889

**Published:** 2017-01-10

**Authors:** Qian Zhang, Xiaofang Sun, Xinhua Xiao, Jia Zheng, Ming Li, Miao Yu, Fan Ping, Zhixin Wang, Cuijuan Qi, Tong Wang, Xiaojing Wang

**Affiliations:** 1 Key Laboratory of Endocrinology, Translational Medicine Centre, Ministry of Health, Department of Endocrinology, Peking Union Medical College Hospital, Peking Union Medical College, Chinese Academy of Medical Sciences, Beijing, China; 2 Department of Endocrinology, the Affiliated Hospital of Qingdao University, Qingdao, Shandong, China; The University of Manchester, UNITED KINGDOM

## Abstract

Maternal undernutrition is linked with an elevated risk of diabetes mellitus in offspring regardless of the postnatal dietary status. This is also found in maternal micro-nutrition deficiency, especial chromium which is a key glucose regulator. We investigated whether maternal chromium restriction contributes to the development of diabetes in offspring by affecting DNA methylation status in liver tissue. After being mated with control males, female weanling 8-week-old C57BL mice were fed a control diet (CON, 1.19 mg chromium/kg diet) or a low chromium diet (LC, 0.14 mg chromium/kg diet) during pregnancy and lactation. After weaning, some offspring were shifted to the other diet (CON-LC, or LC-CON), while others remained on the same diet (CON-CON, or LC-LC) for 29 weeks. Fasting blood glucose, serum insulin, and oral glucose tolerance test was performed to evaluate the glucose metabolism condition. Methylation differences in liver from the LC-CON group and CON-CON groups were studied by using a DNA methylation array. Bisulfite sequencing was carried out to validate the results of the methylation array. Maternal chromium limitation diet increased the body weight, blood glucose, and serum insulin levels. Even when switched to the control diet after weaning, the offspring also showed impaired glucose tolerance and insulin resistance. DNA methylation profiling of the offspring livers revealed 935 differentially methylated genes in livers of the maternal chromium restriction diet group. Pathway analysis identified the insulin signaling pathway was the main process affected by hypermethylated genes. Bisulfite sequencing confirmed that some genes in insulin signaling pathway were hypermethylated in livers of the LC-CON and LC-LC group. Accordingly, the expression of genes in insulin signaling pathway was downregulated. There findings suggest that maternal chromium restriction diet results in glucose intolerance in male offspring through alterations in DNA methylation which is associated with the insulin signaling pathway in the mice livers.

## Introduction

Recently, the incidence of diabetes has dramatically increased, especially type 2 diabetes (T2D). Sedentary lifestyle, insufficient exercise, and high calories food are undoubtedly contributing to the increase of T2D. On the other hand, more and more studies are examining the linkage between the intrauterine environment of the fetus and later health. Increasing evidence in human populations shows that intrauterine growth retardation (IUGR) leads the onset of metabolic diseases in adult life, such as insulin resistance and T2D [1, 2]. In this extremely sensitive window period, some specific variations in the genome “program” the metabolic status in adult life [3, 4]. One of the main “programming” mechanisms is DNA methylation in the fetal period, and this DNA methylation information can transfer to the later life [5, 6].

Chromium (Cr) is considered to be a key glucose regulator. Mertz *et al*. were the first to report the blood glucose regulation function of chromium [7]. Later studies in hospitalized patients showed that chromium supplementation improved glucose tolerance [8, 9]. The minimum suggested daily chromium intake is 30 μg. However, the average dietary chromium intake for adults is far below this recommendation in many countries [10, 11]. In particular, pregnant women and elderly individuals are more prone to the chromium deficiency [12], due to increased metabolic stress and decreased absorption ratio [13, 14]. Our previous study showed that maternal low chromium diet increased body weight in female mice offspring, although the food intake was comparable among different groups [15]. Recent studies have shown that maternal dietary chromium limitation induces insulin resistance and impaired glucose tolerance in WNIN rat offspring. The mechanism is associated with enhanced oxidative stress, which may lead pups to type 2 diabetes in their later life [16].

Here, we used a mouse model of maternal chromium limitation diet to examine whether disruption of the programmed DNA methylation in the liver of offspring links the early nutrition and glucose metabolism disorders in the later life.

## Materials and Methods

### Animals and diets

This study was implemented in accordance with the recommendations of the guidelines of the Ethical Committee for Animal Use of Peking Union Medical College Hospital, who approved the study (Permit Number: MC-07-6004), and all efforts were made to minimize suffering. Sixteen 7-week-old virgin female C57BL mice (18.1 ± 1.4 g) were obtained from the Institute of Laboratory Animal Science, Chinese Academy of Medical Sciences and Peking Union Medical College (Beijing, China, SCXK-2013-0110). After 1 week of adaptation, the female mice were housed with male mice (2:1) overnight for mating. Pregnancy was confirmed by the checking for the presence of vaginal smears, the pregnant female mice (n = 16) were housed in individual cages in a room at 24°C ± 1°C with lights on from 6:00 to 18:00. The pregnant mice were randomly divided into 2 groups, and fed with two types of diet through gestation and lactation: a standard control diet group (CON, 1.19 mg chromium/kg diet) and a low chromium diet group (excluded only in chromium, LC, 0.14 mg chromium/kg diet, n = 8/group). The control diet was prepared according to the American Institute of Nutrition (AIN)-93G formulation and analyzed for chromium content with an atomic absorption spectrometer (TAS986, Beijing Persee General Corporation, Beijing, China) using flame spectrophotography [17]. The low-chromium diet (LC) was prepared by only excluding the chromium salt in the mineral mixture that was added to the diet. All diets were produced by Research Diets (New Brunswick, NJ, USA, S1 and S2 Tables). Pregnant female mice were fed with the specific diet (CON or LC) and water *ad libitum* during the pregnancy and lactation periods. On the first day after birth (d0), the litter sizes of both the groups were adjusted to 6 animals per litter (3 male, 3 female, if possible). After weaning, offspring (3 weeks of age) were sub-grouped into four groups: CON-CON (offspring born from control dams was fed with control diet from weaning), CON-LC (offspring born from control dams was fed with low chromium diet from weaning), LC-CON (offspring born from LC dams was fed with control diet from weaning), and LC-LC (offspring born from LC dams was fed with low chromium diet from weaning, n = 8/group, one male pup from each litter was randomly assigned to each group) until 32 weeks of age. Only male offspring were used for the present study to avoid sex differences in early-life nutrition or glucose metabolism [18]. At the end of the experimental period (32 weeks of age), food was removed for 10 hours, and then the male mice were anesthetized by ketamine (100 mg/kg i.p., Pharmacia and Upjohn Ltd, Crawley, UK, n = 8 per group), and sacrificed by decapitation. Blood samples were collected from intraorbital retrobulbar plexus. The livers of these offspring were quickly removed and stored at -80°C for further analysis.

### Measurement of serum chromium level

Serum chromium was assayed using atomic absorption spectrophotometry (Hitachi, Japan) in the offspring at 32 weeks of age. Each sample was analyzed in duplicate. Strict quality control was performed using standard reference materials (Seronorm^™^ Trace Elements Serum; Nycomed AS, Norway).

### Measurements of body weight and food intake

At birth, each litter was weighed and the average birth weight for each pup was computed as litter weight/litter size. Body weight was assessed again at weaning and 32 weeks of age. At 32 weeks of age, the animals were given a pre-weighed amount of food, and food intake was recorded 24 hours later.

### Measurements of blood glucose and oral glucose tolerance test (OGTT)

At 3 and 32 weeks of age, blood glucose was measured by the glucose oxidase method (Contour TS glucometer, Bayer, Hamburg, Germany). OGTT was performed after feeding deprivation for 10 hours at 32 weeks of age. After collection of a basal sample (0 min), 2 g of glucose/body weight was administered orally. Tail blood samples were collected at 30, 60 and 120 min after glucose administration. The area under the glucose tolerance curve (AUC) was calculated as the integrated area under the curve above the basal value over the 120-min sampling period [19].

### Measurement of insulin and homeostasis model assessment of insulin resistance (HOMA-IR)

At 32 weeks of age, mice were fasted for 10 hours to obtain blood to measure serum insulin. Serum insulin was measured using an ELISA kit (Millipore, Bellerica, MA, USA). HOMA-IR was calculated as insulin (μU/mL) x blood glucose (μU/mL) /22.5 [20].

### Genomic DNA extraction and array hybridization

Genomic DNA (gDNA) was extracted from liver samples using a DNeasy Blood & Tissue kit (Qiagen, Fremont, CA, USA). The quantity and quality of the purified gDNA was then assessed with a Nanodrop ND-1000 (NanoDrop Technologies, Wilmington, DE, USA). The gDNA of each sample was sonicated into 200–1000 bp fragments. Immunoprecipitation of methylated DNA fragments was conducted using Biomag^™^ magnetic beads coupled with mouse monoclonal anti-5-methylcytidine (Diagenode, Liege, Belgium). We labeled the total input and immunoprecipitated DNA with Cy3 and Cy5 fluorophores, respectively. Labeled DNA was then hybridized to the Arraystar Mouse RefSeq Promoter Array, which contains 22,327 well-characterized RefSeq promoter regions (from approximately -1300 to +500 bp from the transcription start site, Agilent Technologies, Waldbronn, Germany), totally covered by ~130,000 probes. Finally, the array was scanned with the Agilent Scanner G2505C (Agilent Technologies, Waldbronn, Germany).

### Data normalization and analysis

Raw data collected from the scanner were normalized and median centered. The results were used in a sliding-window peak-finding algorithm (Roche Diagnostics, GmbH, Mannheim, Germany). To find the significantly positive probes, a one-sided Kolmogorov-Smirnov (KS) test was used (*P*-value<0.01). Then, we calculated M values to compare differences between the two groups (LC-CON group and CON-CON group). M = Average (log2^MeDIP(LC-CON group)/Input(LC-CON group)^)–Average (log2^MeDIP(CON-CON group)/Input(CON-CON group^). Finally we used these data to find the differential methylation-enrichment peaks of genes.

Promoters of the methylation-enriched genes were then grouped into three categories: high CpG promoters (HCPs, with a 500-bp region with a GC fraction ≥ 0.55 and CpG observed/expected ≥ 0.6), low CpG promoters (LCPs, without the 500 bp region in which CpG observed/expected ≥ 0.4), and intermediated CpG promoters (ICPs, with CpG density between HCPs and ICPs) using the dataset generated by Mikkelesen *et al*. [21].

### Pathway and GO analysis

Genes affected by differential expression promoters (DEPs) were analyzed with the DAVID annotation system (http://david.ncifcrf.gov/) for gene ontology and Kyoto Encyclopedia of Genes and Genomes (KEGG, http://www.kegg.jp/) pathway analysis [22].

### Bisulfite DNA sequencing (BSP)

The methylation levels of the selected differentially methylated genes, namely regulating synaptic membrane exocytosis 2 (*Rims2*), harvey rat sarcoma virus oncogene (*Hras1*), thymoma viral proto-oncogene 1 *(Akt1)*, and kirsten rat sarcoma virus oncogene homolog (*Kras*), were determined by BSP. Briefly, 1 microgram of DNA was treated with the EZ DNA Methylation Kit (Zymo Research, Hiss Diagnostics, Germany). The modified DNA was then amplified by PCR with primers designed with the Methyl Primer Express software version 1.0 (Applied Biosystems, Foster City, CA, USA, S3 Table), which corresponded to regions on the microarrays. PCR products were purified using the MinElute Gel Extraction Kit (Invitrogen, Carlsbad, CA, USA) and cloned into the pMD18-T Vector (Takara). The plasmids were purified using the PureLink Miniprep Kit (Invitrogen, Thermo Scientific Inc, Waltham, MA, USA). The positive clones were confirmed by PCR, and no fewer than 10 clones were randomly selected for each mouse for sequencing using an automatic sequencer (ABI Prism 7700 Sequence Detection, Applied Biosystems, Foster City, CA, USA). Sequencing results were analyzed using QUMA (http://quma.cdb.riken.jp/top/quma_main.html) [23].

### Quantitative real time PCR

Total RNA was isolated using the Qiagen RNeasy Mini Kit (Qiagen, Germantown, MD, USA). A Takara reverse transcription kit (Shiga, Japan) was used to make first strand cDNA. The PCR amplification program was as follows: 20 sec at 95°C, followed by 40 cycles at 95°C for 5 sec, and annealing at 60°C for 30 sec in an ABI 7900 Thermocycler (Applied Biosystems, Foster City, CA, USA). The primer sequences are listed in S4 Table. Data analysis was performed using the 2^ΔΔCt^ method. All samples were normalized to *CypA*.

### Statistical analysis

The results are presented as the means ± SD. Data were used by unpaired *t* test and two-way ANOVA. Tukey’s test was used for *post hoc* comparisons. For GO and KEGG pathway analysis, Fisher’s exact test was used. Differences with *P*<0.05 were considered significant. GraphPad Prism software version 5.0 (San Diego, CA, USA) was used to analyze the data.

## Results

### Blood glucose in C57BL mice dams

Fasting blood glucose of the C57BL mice dams were comparable between the CON and LC groups (6.1 ± 1.1 vs. 5.8 ± 1.2 mmol/L, *P*>0.05).

### Body weight, food intake, and serum chromium concentrations in pups

Despite comparable birth and 3-week-old body weights (Table 1) and maintaining a uniform litter size of 6 pups/dam from postnatal day 1, LC-CON, LC-LC, and CON-LC male pups had higher body weights than CON-CON pups at 32 weeks of age (*P*<0.05, Table 1). However, food intake was comparable among the four groups at 32 weeks of age (Table 1). LC-LC and CON-LC offspring had significantly lower serum chromium levels (*P*<0.01) at 32 weeks of age than CON-CON pups. However, LC-CON pups had comparable chromium levels with controls at 32 weeks of age (Table 1).

**Table 1:** Effect of maternal chromium restriction on metabolic variables in male mice offspring.

	Offspring of CON-fed dams		Offspring of LC-fed dams	
Birth weight, g	1.47 ± 0.14		1.40 ± 0.16	
3 weeks of age				
Body weight, g	8.14 ± 1.10		8.56 ± 1.06	
Fasting blood glucose, mmol/L	5.8 ± 0.6		5.6 ± 1.2	
	CON-CON	CON-LC	LC-CON	LC-LC
32 weeks of age				
Body weight, g	31.42 ± 3.86	35.63 ± 4.34*	38.82 ± 3.91*	38.70 ±2.97*
Food intake, g/d	5.38 ± 1.28	5.33 ± 1.95	5.38 ± 1.55	5.21 ± 1.73
Serum chromium, ng/mL	0.83 ± 0.13	0.32 ± 0.06**^&&^	0.73 ±0.11^##^	0.18 ± 0.06**^##^^&&^
Fasting blood glucose, mmol/L	6.1 ± 1.2	8.9 ± 0.5*	8.8 ± 0.9*	8.5 ± 1.1*
Serum insulin, ng/mL	0.36 ± 0.08	0.42 ± 0.04	0.48 ± 0.06*	0.49 ± 0.05*
HOMA-IR	2.08 ± 0.78	4.12 ± 1.56*	4.79 ± 1.04*	4.59 ± 1.32*

Data are expressed as means ± SD (n = 8).

* *P*<0.05,

** *P*<0.01, compared with CON-CON group;

^##^ *P*<0.01 compared with CON-LC group;

^&&^ *P*<0.01, compared with LC-CON group.

CON, control diet; LC, low chromium diet.

### Fasting blood glucose (FBG) in 3- and 32-week-old male pups

At 3 weeks of age, FBG in the LC group was similar to that in the CON group (Table 1); whereas, FBG in the LC-LC group and CON-LC groups was higher than that in the CON-CON group (*P*<0.05) at 32 weeks of age. Returning to normal diet (LC-CON) did not alleviate the elevated blood glucose (*P*<0.05, Table 1).

### Oral glucose tolerance test (OGTT) in male pups

**Fig 1:**
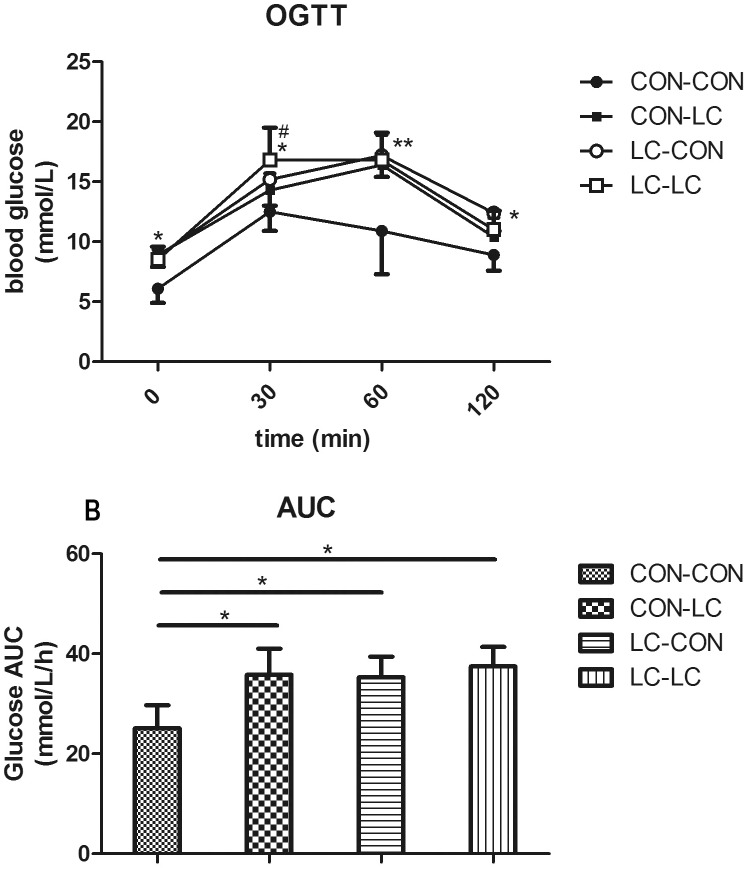
The effect of maternal chromium restriction on glucose tolerance in male pups. A: oral glucose tolerance test (OGTT); B: area under curve (AUC) in OGTT. * *P*<0.05, ** *P*<0.01, compared with CON-CON group; # *P*<0.05 compared with CON-LC group.

At 32 weeks of age, blood glucose levels were significantly higher in the LC-LC, CON-LC, and LC-CON group before (0 min, *P*<0.05) and 30 (*P*<0.05), 60 (*P*<0.01), and 120 (*P*<0.05) min after a bolus gavage of glucose (Fig 1). Consistently, glucose area under curve (AUC) during the OGTT in the LC-CON, LC-LC, and LC-CON group was significantly higher than in the CON-CON group (*P*<0.05, Fig 1). Blood glucose at 30 min after glucose gavage was higher in LC-LC group than that in CON-LC group (*P*<0.05, Fig 1).

### Serum insulin and HOMA-IR in male pups

At 32 weeks of age, serum insulin levels were higher in the LC-LC and LC-CON groups than the CON-CON group (*P*<0.05, Table 1). HOMA-IR was higher in the LC-LC, CON-LC, and LC-CON group than CON-CON group (*P*<0.05, Table 1).

### Methylation array assay

**Fig 2:**
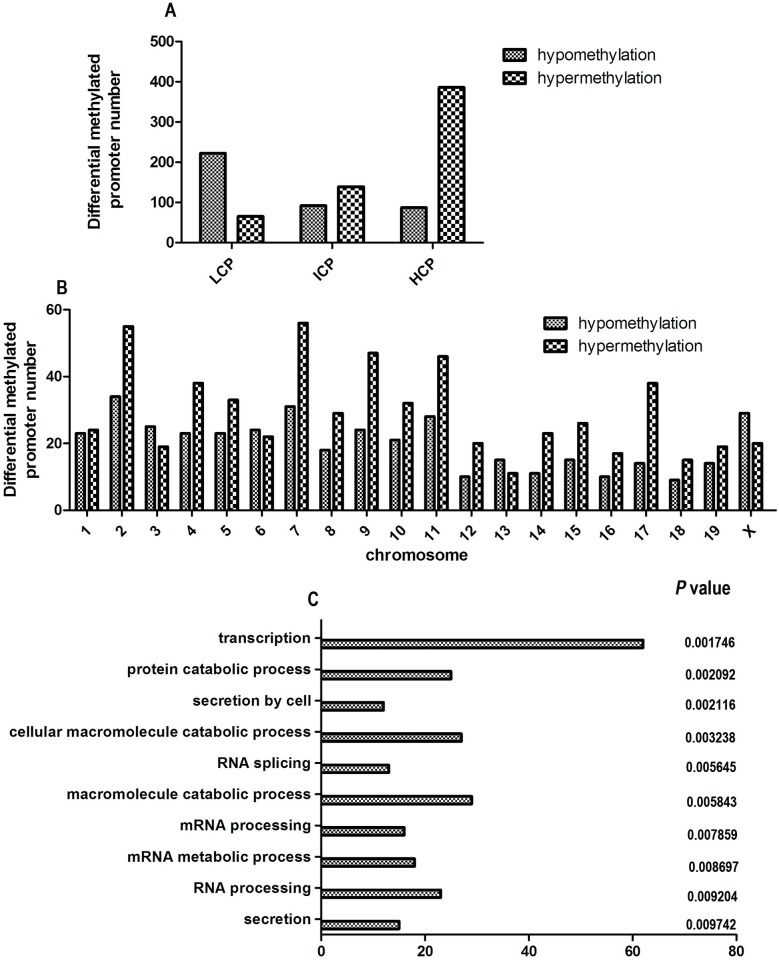
CpG density of differential methylated promoter, the chromosomal distribution of 991 promoter methylated genes, and Gene ontology classifications for differential hypermethylated genes in biology process. A: CpG density of differential methylated promoter; B: the chromosomal distribution of 991 promoter methylated genes; C: Gene ontology classifications for differential hypermethylated genes in biology process. Classification of all promoters with high (HCP), intermediate (ICP), and low (LCP) CpG content. Gene ontology classifications for differential hypermethylated genes in biology process. The GO term is on the Y axis, number of gene is on the X axis, and the *P*-value indicating significance of enrichment is on the right side. GO, gene ontology.

We identified 590 hypermethylated and 401 hypomethylated regions in the LC-CON group compared with the CON-CON group. These promoters belong to 935 genes (560 hypermethylated genes, 375 hypomethylated genes). Among the hypermethylated regions, most were in HCPs (386 regions, 65%, Fig 2A), whereas most hypomethylated regions were in LCPs (55%). The hypermethylated genes were mainly distributed on chromosomes 2, 4, 5, 7, 9, 10, 11, and 17, whereas the hypomethylated genes were mainly on chromosomes 2 and 7 (Fig 2B).

### Annotation of differential DNA methylation of genes

To fully clarify the biological and cellular effects of maternal chromium limitation diet on offspring, we performed gene ontology (GO) analysis of the differentially methylated genes. Because most of the hypermethylated promoters were located in HCPs, we just show the analysis of the hypermethylated promoters here. Hypermethylated genes in the LC-CON group were enriched in the biological processes of secretion (*P* = 0.0017), RNA processing (*P* = 0.0020), mRNA metabolic process (*P* = 0.0021) and so on in biology process (S5 Table, Fig 2C).

### KEGG pathway analysis

**Fig 3:**
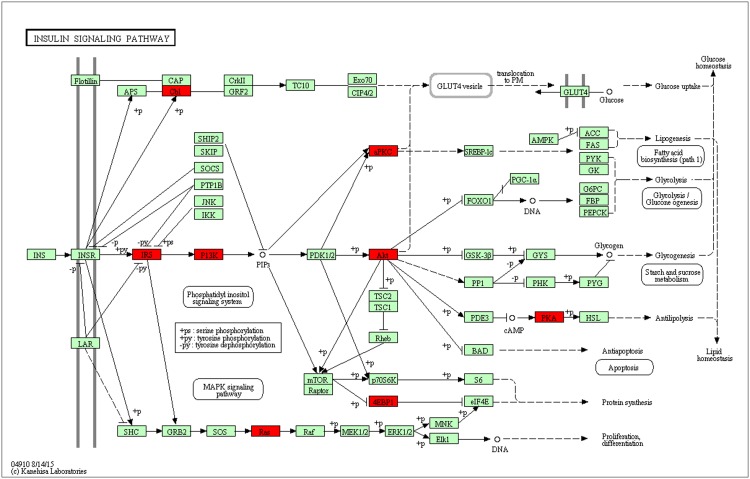
Hypermethylated genes in LC-CON group in the KEGG insulin signaling pathway. The red box indicates hypermethylated gene. CON: control diet; LC, low chromium diet; KEGG, Kyoto Encyclopedia of Genes and Genomes.

The hypermethylated genes were enriched in the insulin signaling pathway (*P* = 0.0125), spliceosome (*P* = 0.0219), Notch signaling pathway (*P* = 0.0259), acute myeloid leukemia (*P* = 0.0394), and ErbB signaling pathway (*P* = 0.0467, S6 Table). Fig 3 and S7 Table show the hypermethylated genes in the LC-CON group involved in the insulin signaling pathway.

### Validation of the methylation level in array results

**Fig 4:**
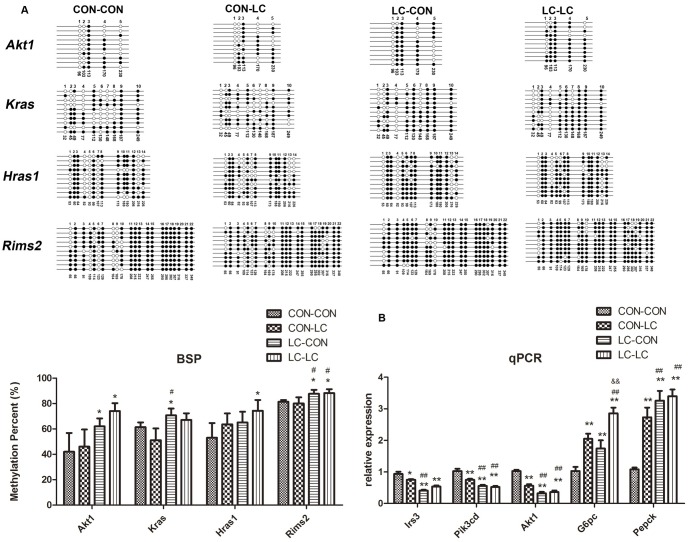
BSP measurements confirm the DNA methylation status of promoter region in the *Akt1*, *Kras*, *Hras1*, and *Rims2* and the relative expression of key genes in insulin signaling pathway. A: BSP measurements confirm the DNA methylation status of promoter region in the *Akt1*, *Kras*, *Hras1*, and *Rims2*; B: the relative expression of key genes in insulin signaling pathway and gluconeogenesis. Black circles indicate methylated CpGs, white circles represent unmethylated CpGs. Values in each bisulfite grouping indicate the percentage of CpG methylation. The data are expressed as mean ± SD (n = 8). For BSP, pooled DNA of eight mice for each group we used for analysis. For each gene in each group, 10 clones randomly selected were analyzed to represent the average methylation level for the indicated genes. * *P*<0.05, ** *P*<0.01, compared with CON-CON group; # *P*<0.05, ## *P*<0.01, compared with CON-LC group; && *P*<0.01, compared with LC-CON group. CON, control diet; LC, low chromium diet. *Rims2*, regulating synaptic membrane exocytosis 2; *Hras1*, harvey rat sarcoma virus oncogene; *Akt1*, thymoma viral proto-oncogene 1; *Kras*, kirsten rat sarcoma virus oncogene homolog; *Irs3*, insulin receptor substrate 3; *Pik3cd*, phosphatidylinositol 3-kinase catalytic delta polypeptide; *G6pc*, glucose-6-phosphatase; *Pepck*, phosphoenolpyruvate carboxykinase.

To verify the results of the methylation array experiments, four candidate promoter-hypermethylated genes were selected and examined by BSP. Because these four genes (regulating synaptic membrane exocytosis 2 *(Rims2*), harvey rat sarcoma virus oncogene (*Hras1*), thymoma viral proto-oncogene 1 (*Akt1*), and kirsten rat sarcoma virus oncogene homolog (*Kras*)) are in the insulin signaling pathway, and their promoters located in HCPs. The methylation levels of *Akt1* and *Rims2* in the LC-CON and LC-LC group were higher than that in the CON-CON group (*P*<0.05). The methylation level of *Hras1* in the LC-LC was higher than that in the CON-CON group (*P*<0.05). The methylation level of *Kras* in the LC-CON group was higher than that in the CON-CON group (*P*<0.05, Fig 4A). The methylation level of *Kras* in the LC-LC group was higher than that in the CON-LC group (*P*<0.05, Fig 4A).

### Real time PCR

The methylation status in the promoter is related to the regulation of gene expression. Thus, we examined the expression of several hypermethylated genes in the insulin signaling pathway and downstream gluconeogenesis. We found that the expression of thymoma viral proto-oncogene 1 (*Akt1*), phosphatidylinositol 3-kinase catalytic delta polypeptide (*Pik3cd*), andinsulin receptor substrate 3 (*Irs3*) was downregulated, however the expression of glucose-6-phosphatase (*G6pc*) and phosphoenolpyruvate carboxykinase (*Pepck*) was upregulated in the CON-LC, LC-CON and LC-LC group than that in CON-CON group (*P*<0.01, Fig 4B). The expression of *Akt1*, *Pik3cd*, and *Irs3* was also lower in LC-LC and LC-CON group than that in CON-LC group (*P*<0.01, Fig 4B).

## Discussion

The fetal environment is correlated with health in later life [24]. Both under and excess nutrition *in utero* can enhance risk of type 2 diabetes in adulthood [1, 25, 26]. The mechanism may involve epigenetic programming such as DNA methylation at CpG islands [27].

Similar to previous reports [28], we found that chromium limitation diet for 7 weeks did not affect their blood glucose, probably indicating that the duration of chromium restriction was insufficient. In addition, chronic maternal chromium limitation did not affect the birth weights and weaning body weight of the offspring. Until 32 weeks of age, body weight in the LC-LC group was higher than that in CON-CON group with comparable food intake among groups. Interesting, reverse diet could not normalize the body weight. We found that the increase in body weight in offspring occurred earlier than observed in other study (12 months of age) [16], possibly because we used a more extreme chromium restriction diet [16].

Moreover, chronic maternal chromium restriction increased blood glucose and the AUC of blood glucose during OGTT in offspring at 32 weeks of age. Previous studies also found glucose metabolism dysfunction at various times (from weaning to 15 months) in offspring of undernourished dams. Padmavathi *et al*. found that maternal chromium restriction increases plasma glucose (from 9 months), and the area under the curve of glucose during oral glucose tolerance test (from 15 months) in the offspring [16]. The offspring of dams fed a low-protein diet have impaired glucose tolerance at weaning [29] and adulthood [30]. At 12 months of age, offspring from vitamin B_12_-restricted dams have higher blood glucose and impaired glucose tolerance [31].

We also found that chronic maternal chromium restriction increased the serum insulin and HOMA-IR. Padmavathi *et al*. found that maternal chromium restriction increases serum insulin (from 15 months) and HOMA-IR (from 9 months) and rehabilitation does not correct this effect [16]. Several studies have found that, although serum insulin does not increase, HOMA-IR increases in offspring from protein-restricted dams at 3 weeks [29] and 3 months [32]. A maternal diet with 50% of the typical digestible energy results in hyperinsulinemia in offspring at postnatal day 160 [33]. From our results, maternal chromium restriction increased body weight, impaired glucose tolerance, and led insulin resistance. Indeed, obesity strongly impairs the glucose tolerance and increases insulin resistance. However, previous study revealed that insulin resistance (from 9 months) showed up earlier than increase of adipose tissue mass (from 18 months) in male rats from maternal chromium restriction[16, 28].

Early nutritional environment may influence the risk of later metabolic disease through alterations of the epigenome, such as changes in DNA methylation and histone modifications. Epigenetics is the study of the modifications in gene expression that are not directly due to variations of the DNA nucleotide sequence and can potentially be transmitted over gaps [34].

DNA methylation occurs mainly at cytosines in CpG islands, and it is a basal epigenetic mark in mammals that regulates the regulation of gene transcription. In mammals, CpG islands are usually located in promoter regions [35, 36]. Highly methylated CpG islands in gene promoter regions can inhibit the gene expression [37].

We found that several genes in the insulin signaling pathway were hypermethylated in the LC-CON group, such as *Akt1*, *Kras*, *Hras1*, and *Rims2*. Especially, the methylation sites of these four genes locate in HCPs. However, in our previous study, no abnormal DNA methylation of key genes in insulin signaling pathway was found in adipose tissue from pup mice born from chromium restriction mothers [15]. This may be due to the tissue specific modification of DNA methylation.

DNA methylation is an important modification in normal and disease development. Growing evidence showed that DNA methylation disorder in some key genes is a linkage between maternal malnutrition and offspring metabolic disorder. Both in a rat model and humans, several genes in the insulin response pathway are differentially methylated in placenta exposed to gestational diabetes [38]. Maternal protein restricted diet reduced the methylation of glucocorticoid receptor (GR) promoter and enhanced GR expression in rat offspring liver. This led increased PEPCK expression and increased gluconeogenesis [39]. Even the individuals who were prenatally exposed to famine during the Dutch Hunger Winter in 1944–45 had less DNA methylation of the IGF2 gene compared with their unexposed same-sex siblings [40]. Our study revealed, for the first time, chromium restriction *in utero* can cause epigenetic changes in offspring liver that will disturb the glucose tolerance and last for a lifetime.

We also found that maternal chromium restriction diet reduced genes expression in insulin signaling pathway (*Akt1*, *Irs3*, and *Pik3cd*) and increased the expression of key molecular in hepatic gluconeogenesis (*G6pc* and *Pepck*). Besides skeletal muscle and adipose tissue, liver is an insulin-sensitive target organ. Hepatic insulin resistance has been considered to be an underlying cause of the metabolic syndrome and type 2 diabetes mellitus [41]. In liver, insulin promotes glucose uptake and suppresses hepatic glucose production by stimulating a cascade of signaling processes initiated by the binding of insulin to extracellular α-subunit of the insulin receptor (IR) on the cellular membrane. In the presence of insulin, IR phosphorylates IRS that are linked to the activation of the PI3K-Akt pathway, which is responsible for most of the metabolic actions of insulin [42]. Hepatic insulin resistance is characterized by a blunted suppression of hepatic glucose production in response to insulin, which is secondary to the impairment of insulin signaling [43].

**Fig 5:**
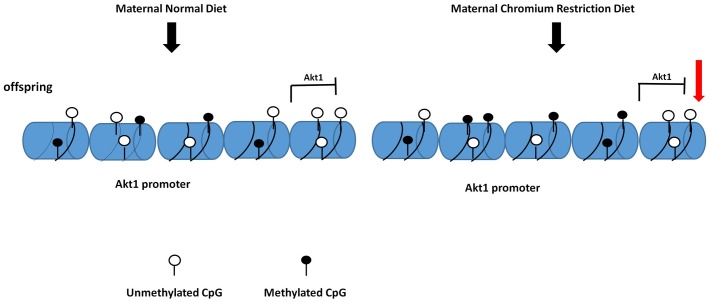
The proposed model for the maternal chromium restriction on DNA methylation modification in *Akt1* promoter, which inhibits *Akt1* expression. *Akt1*: thymoma viral proto-oncogene 1.

Importantly, we found that maternal chromium restriction diet activated methylation of *Akt1* promoters, thus reduced its gene expression. Protein kinase B/Akt belongs to downstream kinases and mediates the effects of insulin on glucose transport, glycogen synthesis and suppression of hepatic gluconeogenesis. Ye *et al*. found hepatocytes in intrauterine growth restriction (IUGR) rats with catch-up growth (CG-IUGR) show decreased *Irs1*, *Pi3k*, and phosphorylated *Akt* expression [44]. In vitro, after transfecting CG-IUGR hepatocytes with suppressor of cytokine signaling 3 (SOCS3, an mediator of insulin signaling)-specific siRNA, protein levels of IRS1, PI3K, and phosphorylated Akt increased, thus insulin signaling transduction and hepatic glucose metabolism was ameliorated. Rapamycin could enhance phosphorylated *Akt* expression in insulin resistance HepG2 cells model [45]. Thus, our results suggest that maternal chromium restriction diet could increase methylation levels of genes in the insulin signaling pathway, thus impair Akt-PI3k signaling, resulting in excess hepatic glucose production which leads to glucose intolerant (Fig 5).

By using a whole genome methylation array, this project aimed to study the modifications in glucose metabolism from chromium restriction of perinatal and new born time window, that may have long-term metabolic disorder consequences. Maternal chromium deficiency during gestation and lactation leads to metabolic inflexibility in the male offspring in later life. This is the first time to report that the involved mechanism is the changes in methylation levels of key gene promoters that impaired insulin signaling. These findings will give us a hint about early intervention to the offspring from undernutrition mothers.

## Supporting Information

S1 Table Composition of diets. CON, control diet; LC: low chromium diet.
(DOCX)

S2 Table Component of minerals mixes in diets. CON, control diet; LC: low chromium diet.
(DOCX)

S3 Table Primers for bisulfite-modified DNA sequencing. *Rims2*, regulating synaptic membrane exocytosis 2; *Hras1*, harvey rat sarcoma virus oncogene; *Akt1*, thymoma viral proto-oncogene 1; *Kras*, kirsten rat sarcoma virus oncogene homolog.
(DOCX)

S4 Table Primers for real time PCR. *Irs3*, insulin receptor substrate 3; *Pik3cd*, phosphatidylinositol 3-kinase catalytic delta polypeptide; *Akt1*, thymoma viral proto-oncogene 1.
(DOCX)

S5 Table Annotation of hypermethylated promoter specific genes from DNA methylation array data in adult male mice offspring liver from maternal chromium restriction programming (*P* value<0.01). (DOCX)

S6 Table KEGG pathway from DNA methylation array data in adult male mice offspring liver from maternal chromium restriction programming (Fold Enrichment>2). KEGG, Kyoto Encyclopedia of Genes and Genomes.
(DOCX)

S7 Table Hypermethylated genes in insulin signaling pathway in adult male mice offspring liver from maternal chromium restriction programming (Peak score>2). *Akt1*, thymoma viral proto-oncogene 1; *Cblc*, casitas B-lineage lymphoma c; *Eif4ebp1*, eukayotic translation initiation factor 4E binding protein 1; *Hras1*, harvey rat sarcoma virus oncogene; *Irs3*, insulin receptor substrate 3; *Kras*, kirsten rat sarcoma virus oncogene homolog; *Pik3cd*, phosphatidylinositol 3-kinase catalytic delta polypeptide; *Prkcz*, protein kinase C, zeta; *Rims2*, regulating synaptic membrane exocytosis 2. TSS, transcription start sites; Peak to TSS, the distance from the center of the peak to the TSS. (“-”: peak center in upstream of the TSS). Peak Score, the average of–log_10_^*P*-value^ from the probes within the peak. The score reflects the probability of positive enrichment. (cut-off = 2). Peak M Value, the median of log_2_^-ratio^ from the probes within the peak. The score reflects the methylation degree.
(DOCX)
