# Supplementary material for: Dietary Chromium Restriction of Pregnant Mice Changes the Methylation Status of Hepatic Genes Involved with Insulin Signaling in Adult Male Offspring
Source: PLoS One. 2017 Jan 10;12(1):e0169889. doi: 10.1371/journal.pone.0169889 (PMC5224989; doi:10.1371/journal.pone.0169889)
Supplement: S1 Table — CON, control diet; LC: low chromium diet. (DOCX) [file pone.0169889.s001.docx]

**S1 Table. Composition of diets.**

| Ingredient (g or mg/kg diet) | CON | LC |
| --- | --- | --- |
| Cornstarch | 397.5 | 397.5 |
| Casein | 200.0 | 200.0 |
| Dextrinized cornstarch | 132.0 | 132.0 |
| Sucrose | 100.0 | 100.0 |
| Soybean oil | 70.0 | 70.0 |
| Fiber | 50.0 | 50.0 |
| Mineral Mix | 35.0 | 35.0 |
| Vitamin Mix AIN-93-VX | 10.0 | 10.0 |
| L-Cystine | 3.0 | 3.0 |
| Choline bitartrate | 2.6 | 2.6 |
| *Tert*-Butylhydroquinone, mg | 14.0 | 14.0 |

CON, control diet; LC: low chromium diet.
