# Supplementary material for: Dietary Chromium Restriction of Pregnant Mice Changes the Methylation Status of Hepatic Genes Involved with Insulin Signaling in Adult Male Offspring
Source: PLoS One. 2017 Jan 10;12(1):e0169889. doi: 10.1371/journal.pone.0169889 (PMC5224989; doi:10.1371/journal.pone.0169889)
Supplement: S2 Table — CON, control diet; LC: low chromium diet. (DOCX) [file pone.0169889.s002.docx]

**S2 Table. Component of minerals mixes in diets.**

| Ingredient (g or mg/kg mix) | CON | LC |
| --- | --- | --- |
| Calcium carbonate anhydrous | 357.00 | 357.00 |
| Potassium phosphate monobasic | 196.00 | 196.00 |
| Potassium citrate, tripotassium monohydrate | 70.78 | 70.78 |
| Sodium chloride | 74.00 | 74.00 |
| Potassium sulfated | 46.60 | 46.60 |
| Magnesium oxide | 24.00 | 24.00 |
| Ferric citrate | 6.06 | 6.06 |
| Zinc carbonate | 1.65 | 1.65 |
| Sodium meta-silicate | 1.45 | 1.45 |
| Manganous carbonate | 0.63 | 0.63 |
| Cupric carbonate | 0.30 | 0.30 |
| Chromium potassium sulfate | 0.28 | 0.00 |
| Boric acid, mg | 81.50 | 81.5 |
| Sodium fluoride, mg | 63.50 | 63.5 |
| Nickel carbonate, mg | 31.80 | 31.8 |
| Lithium chloride, mg | 17.40 | 17.4 |
| Sodium selenite anhydrous, mg | 10.25 | 10.25 |
| Potassium iodate, mg | 10.00 | 10.0 |
| Ammonium paramolydbate | 7.95 | 7.95 |
| Ammonium vanadate, mg | 6.60 | 6.60 |
| Powdered sucrose | 221.03 | 221.03 |

CON, control diet; LC: low chromium diet.
