# Supplementary material for: Dietary Chromium Restriction of Pregnant Mice Changes the Methylation Status of Hepatic Genes Involved with Insulin Signaling in Adult Male Offspring
Source: PLoS One. 2017 Jan 10;12(1):e0169889. doi: 10.1371/journal.pone.0169889 (PMC5224989; doi:10.1371/journal.pone.0169889)
Supplement: S3 Table — Rims2, regulating synaptic membrane exocytosis 2; Hras1, harvey rat sarcoma virus oncogene; Akt1, thymoma viral proto-oncogene 1; Kras, kirsten rat sarcoma virus oncogene homolog. (DOCX) [file pone.0169889.s003.docx]

**S3 Table. Primers for bisulfite-modified DNA sequencing.**

| Gene | Accession number | Primer sequences (from 5’ to 3’) | Production size | CpG number |
| --- | --- | --- | --- | --- |
| *Rims2* | NM_053271 | F: 5’-TTTTTTTTTGTTTTTTGATTGA-3’ | 35 | 22 |
|  |  | R: 5’-ACCCTAAACRAAAAAAAATC-3’ |  |  |
| *Hras1* | NM_001130444 | F: 5’-TTTTGGGTTTTTGGTGAG-3’ | 251 | 14 |
|  |  | R: 5’-TTATAATTAACTAAACAACCCATCC-3’ |  |  |
| *Akt1* | NM_009652 | F: 5’-TTTGGGGAGTTTTTATTAGTTG-3’ | 261 | 5 |
|  |  | R: 5’-TCCAAAACTCCAACTCTAATCT-3’ |  |  |
| *Kras* | NM_021284 | F: 5’-TTGATGGTATTTTTTAGAAGGGGT-3’ | 269 | 10 |
|  |  | R: 5’-AACCTACAAACTCCCAACCC-3’ |  |  |

*Rims2*, regulating synaptic membrane exocytosis 2; *Hras1*, harvey rat sarcoma virus oncogene; *Akt1*, thymoma viral proto-oncogene 1; *Kras*, kirsten rat sarcoma virus oncogene homolog.
