# Supplementary material for: Dietary Chromium Restriction of Pregnant Mice Changes the Methylation Status of Hepatic Genes Involved with Insulin Signaling in Adult Male Offspring
Source: PLoS One. 2017 Jan 10;12(1):e0169889. doi: 10.1371/journal.pone.0169889 (PMC5224989; doi:10.1371/journal.pone.0169889)
Supplement: S4 Table — Irs3, insulin receptor substrate 3; Pik3cd, phosphatidylinositol 3-kinase catalytic delta polypeptide; Akt1, thymoma viral proto-oncogene 1. (DOCX) [file pone.0169889.s004.docx]

**S4 Table. Primers for real time PCR.**

| Gene | Accession number | Primer sequences (from 5’ to 3’) | Production size |
| --- | --- | --- | --- |
| *Irs3* | NM_010571 | F: 5’-GTCATTGTGTCTCCTCAG-3’ | 92 |
|  |  | R: 5’-CTCCTCCCATTGTTTCTG-3’ |  |
| *Pik3cd* | NM_001164050 | F: 5’-TTCATTCTCACCTACGACTT-3’ | 75 |
|  |  | R: 5’-GAACCTTTCAAACTTCTCACT-3’ |  |
| *Akt1* | NM_009652 | F: 5’-CAGTTTGAGACCACACAT-3’ | 75 |
|  |  | R: 5’-GCGTCAGTCCTTAATAGTT-3’ |  |
| *G6pc* | NM_008061 | F: 5’-GAAGGATGGAGGAAGGAA-3’ | 77 |
|  |  | R: 5’-TTGGTAATTCACTTGGAGATAG-3’ |  |
| *Pepck* | NM_ 011044 | F: 5’-CATTGCCTGGATGAAGTT-3’ | 145 |
|  |  | R: 5’-GTTGGTGAAGATGGTGTT-3’ |  |

*Irs3*, insulin receptor substrate 3; *Pik3cd*, phosphatidylinositol 3-kinase catalytic delta polypeptide; *Akt1*, thymoma viral proto-oncogene 1; *G6pc*, glucose-6-phosphatase; *Pepck*, phosphoenolpyruvate carboxykinase.
