# Supplementary material for: Dietary Chromium Restriction of Pregnant Mice Changes the Methylation Status of Hepatic Genes Involved with Insulin Signaling in Adult Male Offspring
Source: PLoS One. 2017 Jan 10;12(1):e0169889. doi: 10.1371/journal.pone.0169889 (PMC5224989; doi:10.1371/journal.pone.0169889)
Supplement: S5 Table — (DOCX) [file pone.0169889.s005.docx]

**S5 Table. Annotation of hypermethylated promoter specific genes from DNA methylation array data in adult male mice offspring liver from maternal chromium restriction programming (*P* value<0.01).**

| Term | Term number | Count | genes | *P* value | Fold enrichment |
| --- | --- | --- | --- | --- | --- |
| Biological process | | | | | |
| secretion | GO:0046903 | 15 | *Pldn, Exoc8, Hps1, Rims2, Amn, Lnpep, Trim36, Syn1, Trim9, Stxbp5, Pou2f2, Rapgef4, Trp73, Pcsk4, Cacna1b* | 0.0017 | 2.6350 |
| RNA processing | GO:0006396 | 23 | *Dhx8, Rpp25, Strap, Magoh, Lsm7, Rrp9, Fdxacb1, Wdr83, Ddx56, Ctu1, Plrg1, Rod1, Larp7, Cdc40, Nudt21, U2af1, Khsrp, Srrm1, Snrnp70, Rbm25, Snrnp25, Luc7l3, Ddx51* | 0.0020 | 2.0433 |
| mRNA metabolic process | GO:0016071 | 18 | *Dhx8, Strap, Magoh, Lsm7, Wdr83, Plrg1, Dnajb11, Rod1, Cdc40, Nudt21, U2af1, Khsrp, Mex3d, Srrm1, Snrnp70, Rbm25, Snrnp25, Luc7l3* | 0.0021 | 2.3139 |
| mRNA processing | GO:0006397 | 16 | *Dhx8, Strap, Magoh, Lsm7, Wdr83, Plrg1, Rod1, Cdc40, Nudt21, Khsrp, U2af1, Srrm1, Snrnp70, Rbm25, Luc7l3, Snrnp25* | 0.0032 | 2.3708 |
| macromolecule catabolic process | GO:0009057 | 29 | *Lsm7, Usp4, Senp8, Dnase1l2, Ube2r2, Akt1, Lnpep, Fbxw7, Ppp2cb, Fbxo42, Ube2d1, Ufd1l, Magoh, Mul1, March6, Brap, March1, Senp3, Pja1, Cblc, Uhrf1, Trim36, Huwe1, Mib2, Med8, Mex3d, Spopl, Asb7, Rnf111* | 0.0056 | 1.7215 |
| RNA splicing | GO:0008380 | 13 | *Wdr83, Dhx8, Plrg1, Strap, Magoh, Cdc40, Lsm7, U2af1, Khsrp, Srrm1, Snrnp70, Snrnp25, Luc7l3* | 0.0058 | 2.5109 |
| cellular macromolecule catabolic process | GO:0044265 | 27 | *Lsm7, Usp4, Senp8, Dnase1l2, Ube2r2, Fbxw7, Ppp2cb, Fbxo42, Ube2d1, Ufd1l, Magoh, Mul1, March6, Brap, March1, Senp3, Pja1, Cblc, Uhrf1, Trim36, Huwe1, Mib2, Med8, Mex3d, Spopl, Asb7, Rnf111* | 0.0078 | 1.7212 |
| secretion by cell | GO:0032940 | 12 | *Pldn, Trim36, Syn1, Exoc8, Trim9, Stxbp5, Pou2f2, Hps1, Rapgef4, Rims2, Pcsk4, Cacna1b* | 0.0086 | 2.504 |
| protein catabolic process | GO:0030163 | 25 | *Usp4, Senp8, Ube2r2, Akt1, Lnpep, Fbxw7, Ppp2cb, Fbxo42, Ube2d1, Ufd1l, Mul1, March6, Brap, March1, Pja1, Cblc, Senp3, Uhrf1, Trim36, Huwe1, Mib2, Med8, Spopl, Asb7, Rnf111* | 0.0092 | 1.7456 |
| transcription | GO:0006350 | 62 | *Mms19, Ppara, Jdp2, Sox21, Zfp41, Arid4b, Maf1, Bhlha15, Olig2, Myst2, Scrt1, Rbpjl, Zfp518b, Zfp553, Rbbp4, Eomes, Ncoa7, Arntl, Cobra1, Hoxc12, Uhrf1, Med8, Zglp1, Khsrp, Mga, Tgif1, Zzz3, Trp73, Supt6h, Litaf, Sox7, Cic, Tcfe3, Dido1, Pou2f2, Per2, Lhx4, Leo1, Rqcd1, Gtf3c2, Nr1h4, Ewsr1, Nat14, Ssrp1, Creb1, Zfp672, Neurog1, Phf12, Tead3, Notch3, Nrf1, Hnrnpul1, Zfp773, Sp5, Irf1, Pprc1, Irf3, Rbpj, Zfp202, Zbtb1, Vps25, Klf3* | 0.0097 | 1.3583 |
| *Cellular Components* | | | | | |
| microtubule | GO:0005874 | 15 | *Kif24, Haus2, Neil2, Katnb1, Birc5, Ptpn20, Kif9, Cenpj, Tubgcp3, Kif1b, Appbp2, Gm16517, Kif21a, Tubb1, Katnal1* | 0.0026 | 2.5128 |
| spliceosome | GO:0005681 | 10 | *Wdr83, Dhx8, Plrg1, Strap, Magoh, Cdc40, U2af1, Srrm1, Snrnp70, Snrnp25* | 0.0036 | 3.2424 |
| ribonucleoprotein complex | GO:0030529 | 22 | *Dhx8, Rpl18, Noc4l, Strap, Magoh, Lsm7, Rps15a, Rrp9, Rpl28, Wdr83, Rps28, Plrg1, Hnrnpul1, Larp7, Cdc40, Rps15, U2af1, Srrm1, Dazl, Snrnp70, Rps21, Snrnp25* | 0.0055 | 1.9145 |
| spindle | GO:0005819 | 9 | *Akt1, Tubgcp3, Haus2, Neil2, Ppp2cb, Katnb1, Birc5, Racgap1, Lats2* | 0.0056 | 3.3197 |
| *Molecular Function* | | | | | |
| zinc ion binding | GO:0008270 | 72 | *Ppara, Solh, Zbtb34, Zfp41, Helz, Mmp23, Lnpep, Dgcr2, Trim9, Nsmce1, U2af1, Scrt1, Myst2, Nt5e, Zfp518b, Zfp553, Neil2, Brap, Zbtb25, Hagh, Pja1, Uhrf1, Trim36, Taf15, Zfp280b, Mib2, Zglp1, Zzz3, Trp73, Zadh2, Adam12, Adamts18, Prkcz, Zfand3, Adamts19, Zbtb10, Amz2, Rims2, Myo9a, Dido1, Plagl1, Ace, Trim68, Lhx4, Rbm27, Nr1h4, Ewsr1, Recql4, Zmym3, Zfp672, Mul1, Trim25, Birc5, Phf12, March6, Racgap1, Dnpep, March1, Msl2, Cblc, Adam1b, S100b, Zfp773, Sp5, Dpm1, Mex3d, Cars2, Cit, Zbtb1, Zfp202, Klf3, Rnf111* | 0.0028 | 1.3855 |
| cytoskeletal protein binding | GO:0008092 | 21 | *Mtss1, Maea, Tnnc1, Wasf1, Neil2, Vil1, Birc5, Fhdc1, Racgap1, Cenpj, Myo9a, Farp1, Rab11fip5, Kif1b, S100b, Syn1, Mib2, Capg, Appbp2, Myo5c, Snta1* | 0.0032 | 2.0549 |
| transition metal ion binding | GO:0046914 | 85 | *Ppara, Solh, Zbtb34, Zfp41, Nup188, Helz, Mmp23, Lnpep, Ndufs7, Dgcr2, Trim9, Nsmce1, P4ha3, U2af1, Scrt1, Myst2, Nt5e, Zfp518b, Zfp553, Leprel2, Neil2, Cyp26a1, Brap, Zbtb25, Ogfod1, Hagh, Pja1, Uhrf1, Ado, Trim36, Taf15, Zfp280b, Ppm1k, Mib2, Zglp1, Zzz3, Trp73, Zadh2, Adam12, Adamts18, Prkcz, Zfand3, Adamts19, Zbtb10, Amz2, Rrm2b, Rims2, Dido1, Myo9a, Plagl1, Ace, Trim68, Ppp2cb, Lhx4, Rbm27, Ewsr1, Nr1h4, Cyp19a1, Recql4, Scd1, Zmym3, Mul1, Zfp672, Birc5, Trim25, Phf12, March6, Racgap1, Dnpep, March1, Msl2, Cblc, Cyp2a22, S100b, Adam1b, Zfp773, Sp5, Dpm1, Mex3d, Cars2, Cit, Zbtb1, Zfp202, Klf3, Rnf111* | 0.0040 | 1.3203 |
