# Supplementary material for: Dietary Chromium Restriction of Pregnant Mice Changes the Methylation Status of Hepatic Genes Involved with Insulin Signaling in Adult Male Offspring
Source: PLoS One. 2017 Jan 10;12(1):e0169889. doi: 10.1371/journal.pone.0169889 (PMC5224989; doi:10.1371/journal.pone.0169889)
Supplement: S6 Table — KEGG, Kyoto Encyclopedia of Genes and Genomes. (DOCX) [file pone.0169889.s006.docx]

**S6 Table. KEGG pathway from DNA methylation array data in adult male mice offspring liver from maternal chromium restriction programming (Fold Enrichment>2).**

| Pathway name | Term ID | count | genes | *P* value | Fold enrichment |
| --- | --- | --- | --- | --- | --- |
| Insulin signaling pathway | mmu04910 | 9 | *Akt1, Prkcz, Cblc, Prkar2a, Eif4ebp1, Irs3, Kras, Pik3cd, Hras1* | 0.0126 | 2.8566 |
| Spliceosome | mmu03040 | 8 | *Dhx8, Plrg1, Magoh, Cdc40, Lsm7, U2af1, Snrnp70, Rbm25* | 0.0219 | 2.8259 |
| Notch signaling pathway | mmu04330 | 5 | *Notch3, Dll4, Jag1, Rbpj, Rbpjl* | 0.0259 | 4.3801 |
| Acute myeloid leukemia | mmu05221 | 5 | *Akt1, Eif4ebp1, Kras, Pik3cd, Hras1* | 0.0394 | 3.8422 |
| ErbB signaling pathway | mmu04012 | 6 | *Akt1, Cblc, Eif4ebp1, Kras, Pik3cd, Hras1* | 0.0467 | 3.0207 |

KEGG, Kyoto Encyclopedia of Genes and Genomes
