# Supplementary material for: Dietary Chromium Restriction of Pregnant Mice Changes the Methylation Status of Hepatic Genes Involved with Insulin Signaling in Adult Male Offspring
Source: PLoS One. 2017 Jan 10;12(1):e0169889. doi: 10.1371/journal.pone.0169889 (PMC5224989; doi:10.1371/journal.pone.0169889)
Supplement: S7 Table — Akt1, thymoma viral proto-oncogene 1; Cblc, casitas B-lineage lymphoma c; Eif4ebp1, eukayotic translation initiation factor 4E binding protein 1; Hras1, harvey rat sarcoma virus oncogene; Irs3, insulin receptor substrate 3; Kras, kirsten rat sarcoma virus oncogene homolog; Pik3cd, phosphatidylinositol 3-kinase catalytic delta polypeptide; Prkcz, protein kinase C, zeta; Rims2, regulating synaptic membrane exocytosis 2. TSS, transcription start sites; Peak to TSS, the distance from the center of the peak to the TSS. (“-”: peak center in upstream of the TSS). Peak Score, the average of–log10P-value from the probes within the peak. The score reflects the probability of positive enrichment. (cut-off = 2). Peak M Value, the median of log2-ratio from the probes within the peak. The score reflects the methylation degree. (DOCX) [file pone.0169889.s007.docx]

**S7 Table.** **Hypermethylated genes in insulin signaling pathway in adult male mice offspring liver from maternal chromium restriction programming (Peak score>2).**

| Gene name | accession | chromosome | Peak start | Peak end | Peak length | Peak to TSS^3^ | strand | Promoter classification | Peak Score^4^ | Peak M Value^5^ |
| --- | --- | --- | --- | --- | --- | --- | --- | --- | --- | --- |
| *Akt1* | NM_009652 | chr12 | 113912609 | 113913286 | 677 | -460 | - | HCP | 2.26 | 0.257 |
| *Cblc* | NM_001161844 | chr7 | 20381718 | 20382773 | 1055 | -87 | - | ICP | 3.18 | 0.199 |
| *Eif4ebp1* | NM_007918 | chr8 | 28370468 | 28370925 | 457 | -101 | + | ICP | 2.78 | 0.073 |
| *Hras1* | NM_001130444 | chr7 | 148379550 | 148380384 | 834 | -64 | - | HCP | 2.8 | 0.199 |
| *Irs3* | NM_010571 | chr5 | 138086444 | 138087307 | 863 | 66 | - | LCP | 3.42 | 0.071 |
| *Kras* | NM_021284 | chr6 | 145198961 | 145199536 | 575 | -497 | - | HCP | 2.07 | 0.180 |
| *Pik3cd* | NM_001164050 | chr4 | 149073444 | 149073716 | 272 | -763 | - | ICP | 2.62 | 0.141 |
| *Prkcz* | NM_001039079 | chr4 | 154719618 | 154719865 | 247 | 139 | - | LCP | 2.33 | 0.050 |
| *Rims2* | NM_053271 | chr15 | 39028542 | 39028787 | 245 | -1212 | + | HCP | 2.35 | 0.099 |

*Akt1*, thymoma viral proto-oncogene 1; *Cblc,* casitas B-lineage lymphoma c; *Eif4ebp1,* eukayotic translation initiation factor 4E binding protein 1; *Hras1*, harvey rat sarcoma virus oncogene; *Irs3*, insulin receptor substrate 3; *Kras*, kirsten rat sarcoma virus oncogene homolog; *Pik3cd*, phosphatidylinositol 3-kinase catalytic delta polypeptide; *Prkcz,* protein kinase C, zeta; *Rims2*, regulating synaptic membrane exocytosis 2.

TSS, transcription start sites; Peak to TSS, the distance from the center of the peak to the TSS. (“-”: peak center in upstream of the TSS).

Peak Score, the average of –log_10_*^P^*^-value^ from the probes within the peak. The score reflects the probability of positive enrichment. (cut-off=2). Peak M Value, the median of log_2_^-ratio^ from the probes within the peak. The score reflects the methylation degree.
